# A Slicer-Independent Framework for Measuring G-Code Accuracy in Medical 3D Printing

**DOI:** 10.3390/jimaging12010025

**Published:** 2026-01-04

**Authors:** Michel Beyer, Alexandru Burde, Andreas E. Roser, Maximiliane Beyer, Sead Abazi, Florian M. Thieringer

**Affiliations:** 1 Department of Oral and Cranio-Maxillofacial Surgery, University Hospital Basel, 4031 Basel, Switzerland; michel.beyer@usb.ch (M.B.); andreas.roser@unibas.ch (A.E.R.); sead.abazi@usb.ch (S.A.); florian.thieringer@usb.ch (F.M.T.); 2 Medical Additive Manufacturing Research Group (Swiss MAM), Department of Biomedical Engineering, University of Basel, 4123 Allschwil, Switzerland; 3 Department of Dental Technology, Faculty of Nursing and Health Sciences, Iuliu Hatieganu University of Medicine and Pharmacy, 400012 Cluj-Napoca, Romania; 4 Faculty of Medicine, University of Zürich, 8032 Zürich, Switzerland; maximiliane.beyer@uzh.ch

**Keywords:** G-code, image processing, CAD/CAM, 3D printing, volume rendering

## Abstract

In medical 3D printing, accuracy is critical for fabricating patient-specific implants and anatomical models. Although printer performance has been widely examined, the influence of slicing software on geometric fidelity is less frequently quantified. The slicing step, which converts STL files into printer-readable G-code, may introduce deviations that affect the final printed object. To quantify slicer-induced G-code deviations by comparing G-code-derived geometries with their reference STL modelsTwenty mandibular models were processed using five slicers (PrusaSlicer (version 2.9.1.), Cura (version 5.2.2.), Simplify3D (version 4.1.2.), Slic3r (version 1.3.0.) and Fusion 360 (version 2.0.19725)). A custom Python workflow converted the G-code into point clouds and reconstructed STL meshes through XY and Z corrections, marching cubes surface extraction, and volumetric extrusion. A calibration object enabled coordinate normalization across slicers. Accuracy was assessed using Mean Surface Distance (MSD), Root Mean Square (RMS) deviation, and Volume Difference. MSD ranged from 0.071 to 0.095 mm, and RMS deviation from 0.084 to 0.113 mm, depending on the slicer. Volumetric differences were slicer-dependent. PrusaSlicer yielded the highest surface accuracy; Simplify3D and Slic3r showed best repeatability. Fusion 360 produced the largest deviations. The slicers introduced geometric deviations below 0.1 mm that represent a substantial proportion of the overall error in the FDM workflow.

## 1. Introduction

Accurate and reliable fabrication is essential in medical 3D printing, particularly for applications involving patient-specific implants, surgical planning models, and anatomical replicas. In these contexts, dimensional fidelity and geometric consistency are critical not only for ensuring proper clinical function but also for satisfying regulatory requirements. For instance, deviations in implant geometry or surgical guide structure may compromise surgical precision, patient safety, or functional integration [1]. Consequently, each stage of the additive manufacturing workflow must be validated to ensure traceability, reproducibility, and clinical reliability [2].

The technical performance of additive manufacturing technologies such as fused deposition modeling (FDM), stereolithography (SLA), and selective laser sintering (SLS) has been extensively investigated [3,4,5,6,7]. However, the influence of slicing software, the component responsible for converting digital models into machine-executable instructions, remains comparatively underexplored. This is especially relevant in medical applications, where surface meshes derived from computed tomography (CT), or magnetic resonance imaging (MRI) must be translated into printed geometries with sub-millimetric precision. Any inaccuracies introduced during slicing are embedded in the G-code and will be reproduced by the printer, independent of mechanical calibration or nominal resolution.

Slicing software processes triangulated surface meshes, typically in STL or 3MF format, and generates G-code that directs the printer’s movements and extrusion behavior [8,9,10]. This process includes mesh repair, model orientation [10,11], planar segmentation, and the calculation of toolpaths for perimeters, infill structures, and supports [9,12,13]. The resulting G-code comprises motion instructions such as G0 and G1, which define positions along the X, Y, and Z axes and regulate material extrusion via the E parameter, while the travel speed is defined by the F parameter [8,14]. These commands are executed layer by layer, producing a physical object that closely conforms to the encoded instructions.

Initially standardized under ISO 6983, G-code remains the primary instruction format for operating FDM-based 3D printers [15]. Despite its structural simplicity, it fully defines the geometric and material deposition parameters of a print, including path sequencing, retraction points, and extrusion volumes. Therefore, the quality and precision of the G-code have a direct effect on the dimensional accuracy of the final object [16]. Nevertheless, G-code is often regarded as an opaque intermediate representation, and its geometric fidelity is rarely evaluated in academic research or regulatory contexts [8,12]. G-code generation depends on slicer-specific algorithms that convert mesh geometries into print instructions according to user-defined settings, including layer height, infill density, print speed, extrusion temperature, and support strategies [12,13,17]. These parameters directly influence the final encoded geometry and may result in measurable variations in both dimensional accuracy and mechanical properties [10,11,13,17]. One example of software-specific behavior is toolpath normalization, where the slicer resamples the mesh to match its internal resolution or path-planning constraints. While intended to optimize motion smoothness or computational efficiency, normalization can introduce deviations from the source geometry that differs significantly between slicers. Since this step precedes physical printing, any deviations are already encoded in the G-code and should be isolated when comparing slicers independent of thermal or hardware effects. Given that many FDM printers rely on third-party slicing software, user choices and software-specific behaviors can impact the precision of the printed object, independent of the printer’s intrinsic capabilities [16,17].

Recent studies have begun to quantify how slicer software directly shapes the geometry encoded in G-code. Matúš et al. [18] compared a proprietary G3DMaker slicer with the universal Simplify3D and, under identical print settings, found that G3DMaker produced protrusions on cylindrical surfaces while Simplify3D yielded irregularities at nozzle transitions. Petruse et al. [19] examined how slicer algorithms divide a model into layers and noted that when the feature height does not evenly divide by the layer thickness, slicers may omit or compress the final fraction of a layer, creating height errors of up to 0.33 mm. Grgić et al. [20] emphasized the importance of slicer-specific settings, such as horizontal expansion, hole compensation and linear advance, in achieving dimensional fidelity.

Because slicer outputs are enacted through G-code, several authors have focused on analyzing and reconstructing geometry from the code itself. Hachimi et al. [21] described G-code as a sequence of movement and extrusion commands and observed that dialects differ among printers and slicers. They developed a program that converts G-code into finite element models and calibrates the virtual filament cross-section, reducing thickness errors to about 1%. Bacciaglia et al. [22] noted the absence of a standard procedure to recover the real geometry after slicing. Their method reads Cura-generated G-code, flags deposition (G1), travel (G0) and layer-change commands, and uses the resulting tool-path coordinates to rebuild a solid CAD model for accurate finite element analysis.

Viewing G-code as low-level assembly, He et al. [23] argued that the instructions capture the combined effect of the CAD design, slicer choice and slicing parameters. They observed that slicer heuristics can produce inaccurate G-code and that differences in how slicers generate toolpaths can lead to divergent geometries. Taken together, these studies show that slicer algorithms and their G-code outputs materially affect dimensional fidelity in FDM. Furthermore, recent work has also used machine-learning methods to optimize infill patterns and detect anomalies directly in G-code sequences [24].

The present study addresses this gap by introducing a slicer-independent and reproducible method for evaluating G-code accuracy in the context of clinically relevant anatomical models. In order to isolate slicer-induced geometric deviation, all process parameters (nozzle diameter, layer height, extrusion width, and print speed) were held constant. Physical printing was not performed, as the objective was to quantify geometric distortion introduced prior to material deposition, independent of thermomechanical influences.

In contrast to previous work that focused mainly on theoretical reconstructions or single case studies, our framework reconstructs both surface and volume from G-code by combining extrusion data, toolpaths, and a calibration object. The calibration object enables coordinate normalization, facilitating direct comparison between G-code derived geometries and the original STL files. This allows for direct, quantitative comparisons between STL reference models and G-code derived prints across multiple slicers. By applying both surface-based and volumetric metrics to quantify slicing-induced deviations, the proposed pipeline enables a standardized and comparative evaluation of slicing-induced distortions, establishing a foundation for more rigorous quality assurance and verification protocols in high-precision clinical 3D printing. By directly evaluating G-code accuracy at the slicing stage, this work quantifies a previously under-reported source of error in medical additive manufacturing and links it to practical quality-control metrics.

## 2. Materials and Methods

This study evaluated the G-code accuracy of five widely used slicing engines using a dataset of twenty mandibular models. This section describes the generation of G-code for each slicer, the reconstruction of point clouds and STL models from the G-code, and the subsequent comparison with the reference meshes. All processing steps were implemented in Python (version 3.8.10).

### 2.1. Slicing Software

The slicing software used in this study are the following: PrusaSlicer (version 2.9.1.; PrusaSlicer Research a.s., Prague, Czech Republic), Cura (version 5.2.2.; Ultimaker B.V., Geldermalsen, The Netherlands), Simplify3D (version 4.1.2.; Simplify3D, Cincinnati, USA), Slic3r (version 1.3.0., developed by Alessandro Ranellucci, Italy) and Fusion 360 (version 2.0.19725; Autodesk GmbH, Munich, Germany). All slicers were configured with identical parameters to ensure comparable toolpath generation across engines. The layer height was set to 0.20 mm and the nozzle diameter to 0.40 mm. Each model was sliced with two perimeters, two rectilinear top and bottom layers, and 100% rectilinear infill. Supports and brims were disabled, and acceleration control was turned off for all engines. The extrusion width was set to 0.40 mm, with an extrusion speed of 80 mm s^−1^ and a travel speed of 130 mm s^−1^. The infill–perimeter overlap was maintained at 55%, and the bridge and gap-fill speeds were set to 60 mm s^−1^ and 20 mm s^−1^, respectively. All slicers used PLA as the reference material profile.

To maintain deterministic slicing conditions, all randomization and adaptive features were disabled whenever available, including random Z-seam placement, random infill seed generation, adaptive layer height, and “optimize wall flow” heuristics. Residual non-determinism arising from internal slicer optimizers cannot be fully deactivated and is acknowledged as a methodological limitation.

The tessellation tolerance (STL export resolution) was set to 0.0125 mm for PrusaSlicer, Cura, Simplify3D, and Slic3r, and to 0.002 mm for Fusion to ensure equivalent mesh fidelity. Fusion 360 enforces a finer STL tolerance (0.002 mm), whereas the other slicers accept 0.0125 mm. Because each slicer processes its respective input mesh natively, comparisons reflect slicer-induced deviations rather than differences in mesh fidelity.

Repeatability tests were restricted to a single machine and material profile per slicer to isolate slicer algorithm behavior. Variations across printer profiles or material presets were not included to avoid introducing confounding factors.

These standardized configurations guaranteed reproducible and comparable slicing outputs across all five software packages, providing a consistent computational foundation for subsequent point-cloud and surface-accuracy analyses.

### 2.2. Data Preparation

The dataset contained 20 segmented mandibles in Standard Tessellation Language (STL), obtained from 20 CT images from a publicly available dataset containing 627 Digital Imaging and Communications in Medicine (DICOM) files from head and neck squamous cell carcinoma (HNSCC) patients at MD Anderson Cancer Center. The databank is offered by “The Cancer Imaging Archive” (TCIA) [25,26,27,28]. Although these CT scans originate from oncological cases, the mandibles selected for analysis did not present osteolytic or reconstructive alterations in the evaluated regions. Thus, the overall bone morphology was preserved and representative of normal anatomical variation. The data acquisition took place between 2003 and 2013, however, all selected scans met a minimum in-plane pixel resolution of ≤0.5 mm and slice thickness ≤ 1.25 mm, which is comparable to current diagnostic imaging protocols used for craniofacial applications. Segmentation was performed in Mimics Innovation Suite (Version 25.0, Materialise NV, Leuven, Belgium) by using a global threshold of 300–3000 Hounsfield Units (HU) followed by 3D region growing and morphological smoothing with a 2-voxel kernel. The final STL surface was exported at a tolerance of 0.01 mm and further refined in 3-Matic 18.0 to eliminate non-manifold edges and holes prior to slicing. Two raters (4 and 7 years’ experience) reviewed each mask; disagreements were resolved by consensus.

**Figure 1:**
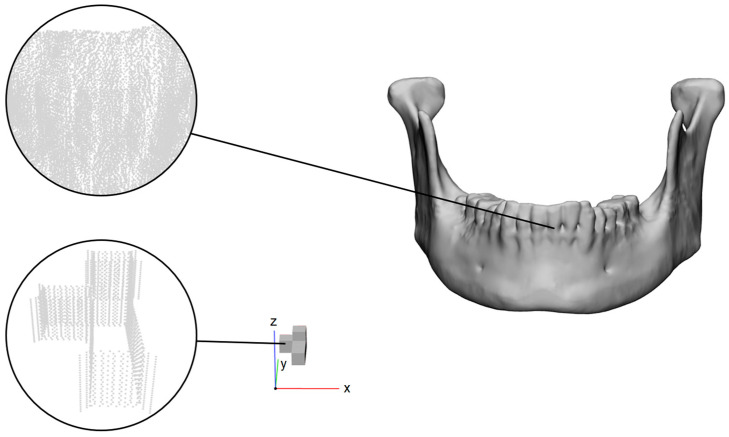
Mandible with its calibration object near the global origin and the corresponding regions as point clouds.

The mandibles were reoriented so that the mandibular border is lying on the XY-plane. Since every slicer uses its own coordinate system, there is the need to register the sliced objects to the original mesh after the slicing process. For this reason, in order not to add any inaccuracies, a calibration object located near the global origin was added to each mandible mesh before the slicing (Figure 1). The object consisted of two orthogonal rectangular blocks forming a step-like shape, with overall bounding dimensions of approximately 10.00 × 6.25 × 8.75 mm^3^ (X × Y × Z). Its three mutually perpendicular faces were aligned with the global coordinate axes (X, Y, Z), providing easily identifiable corners for rigid registration. The calibration object was integrated into the same STL file as the mandibular model, ensuring consistent orientation and identical export across slicers. Subsequently, all mandibles were sliced together with the calibration object as a single unit in each slicing software, and the corresponding G-code was exported.

### 2.3. G-Code to Point Cloud

In order to assess the accuracy of the G-code in comparison to the original mesh, the G-code was transformed into a point cloud (PC1), which is a set of points (Figure 1). The G-code, which consists of printing commands, was read line after line and the points extracted, where the nozzle is extruding (parameter E > 0). This resulted in the raw nozzle centerline point cloud (PC_1_) representing the exact motion path of the printhead. Each extrusion command (G1) was interpreted as a directed segment defined by its start and end coordinates (*x*1, *y*1, *z*1→*x*2, *y*2, *z*2). From the slicer header and line commands, the line width, layer height, and extrusion multiplier were automatically extracted. The reconstruction workflow progressed through four sequential stages: the raw nozzle centerline (PC_1_) obtained directly from G1 motion commands; the XY offset–corrected cloud (PC_2_), adjusted by the negative nozzle radius (−r_nozzle) to approximate the true outer filament boundary; the Z-layer–corrected model (PC_3_), aligning layer heights with the actual deposition plane; and finally the voxelized and meshed surface (PC_4_), yielding a watertight reconstruction suitable for geometric comparison.

Next, PC1 was voxelized into a 3D binary label map (voxel size = 0.10 mm; 26-connectivity), in which occupied voxels represent deposited material. Voxelization at 0.10 mm introduces a quantization uncertainty of approximately ±0.05 mm. While this may smooth high-frequency features, the voxel resolution remained sufficiently fine to preserve slicer-dependent deviations, which exceeded this threshold. Exploratory voxelization tests performed at 0.05 mm on two representative models showed MSD differences below 0.005 mm and preserved slicer ranking, indicating that a 0.10 mm grid provides adequate resolution for comparative analysis.

**Figure 2:**
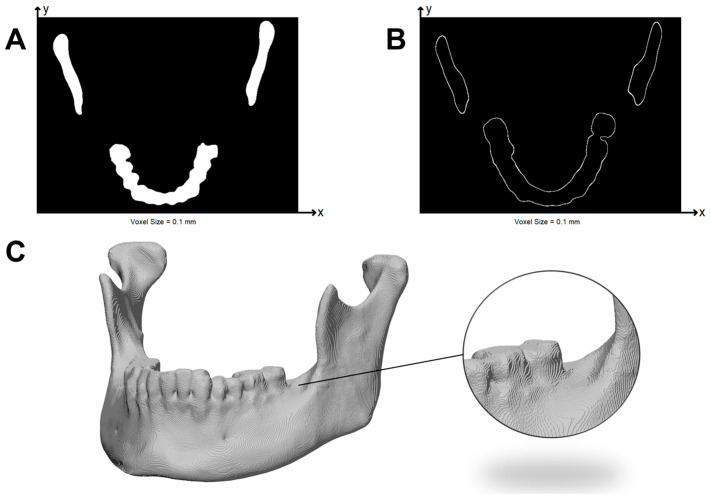
Voxel-based representation of the mandible after marching cubes reconstruction. (**A**) Axial view of the complete voxelized mandible. (**B**) Axial view showing only the voxels corresponding to the outer shell. (**C**) Three-dimensional rendering of the voxelized mandible.

From this binary volume, a watertight surface was extracted using the marching cubes algorithm. Small voids were closed by morphological operations, and only the voxels corresponding to the outer shell of the object were retained. Infill points or inner perimeters were removed, as they do not influence surface or volumetric accuracy. This newly shell-only point cloud is called PC2. An example of the reconstructed volume and the axial view of the 3D binary label of the outer voxels are shown in Figure 2.

### 2.4. Point Cloud XY-Correction

**Figure 3:**
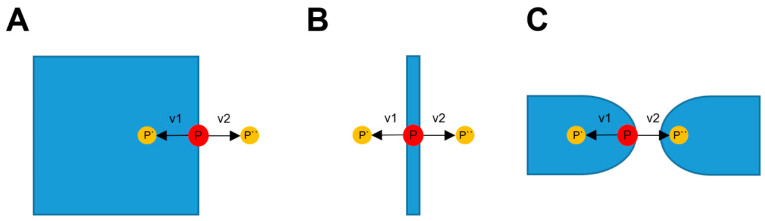
XY point-correction cases based on infill detection. For each point P, two offset candidates P′ and P″ are generated along the normal directions v1 and v2. (**A**) One candidate lies inside the infill and the other outside; the outer point is selected. (**B**) Both candidates lie outside the infill; both are retained. (**C**) Both candidates lie inside the infill; no point is retained.

The points in PC2 represent the nozzle-center positions. The nominal nozzle diameter was 0.40 mm, but the effective line width (w) used for perimeter generation was extracted directly from each slicer’s configuration file. Accordingly, the XY offset applied to each extrusion path was set to −w/2, representing the lateral expansion of deposited material beyond the nozzle centerline. Because the nozzle diameter is 0.40 mm, points were offset by 0.20 mm along the local normal direction (along the normal of the tangent and at the same Z-height) to account for the actual extrusion path, as the deposited material extends beyond the nozzle center by half its diameter. The tangent was calculated, by detecting for each point P in PC2, the previous and the following point defined in the G-code. Subsequently, the two normal 2D vectors (v1 and v2) to the tangent were calculated. Irregular point spacing may introduce noise in tangent estimation; however, subsequent voxel-based shell filtering suppresses these oscillations, preventing them from propagating to the reconstructed geometry. For each vector, two new points were determined (P′ = P + v1, P″ = P + v2), both having the same Z-coordinate as the original point. It was then checked whether these points were located inside the infill of the mesh. This was performed with the 3D binary label map, which was previously generated. If a point is located inside a voxel, which is not part of the outer shell, it is considered to be inside the infill. The possible outcomes of the XY-offset evaluation are summarized in Table 1. The point correction in the XY-plane for different cases is displayed in Figure 3. The point cloud of these corrected points is called PC3.

No explicit smoothing filter was applied to the tangent or normal vectors. However, voxel-based shell filtering and the selection logic between candidate normals minimize oscillatory behavior on curved surfaces.

The correctness of the XY- and Z-corrections was verified independently of the STL reference by evaluating the spatial consistency between corrected points and the voxelized G-code volume. XY-corrected points were required to lie on boundary voxels, ensuring that no corrected point intruded into infill regions. For Z-correction, corrected points were checked against slicer-defined layer-height commands, confirming that >99.9% of points aligned within ±0.10 mm of the expected deposition plane.

### 2.5. Point Cloud Z-Correction

**Figure 4:**
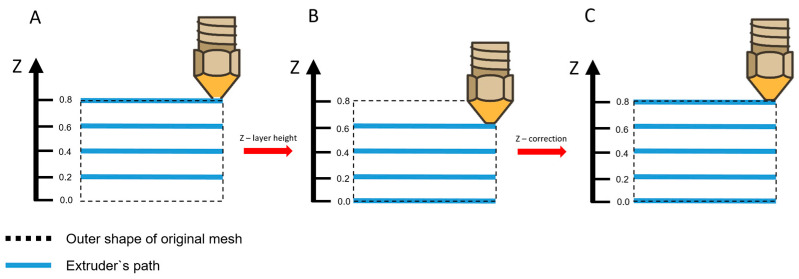
Point correction in the XZ/YZ-plane. (**A**) Extruder’s path in the original G-code. (**B**) Extruder’s path after moving each point down by the layer height. (**C**) Extruder’s path after applying the Z-Correction.

The points in PC3 were already corrected in the XY-direction, but also the Z-coordinate needs to be adapted. The nozzle position in G-code defines the nozzle tip height, whereas the centroid of the deposited filament lies approximately one layer height below this position. Therefore, each point was translated along the *Z*-axis by the local layer height (h) to align the reconstructed geometry with the actual deposition surface. The nozzle’s Z-position is above the true position by a distance corresponding to the layer height, hence all points need to be moved down along the *Z*-axis by exactly this distance. Subsequently, for each point the corresponding voxel in the previously generated 3D binary label map was extracted. It was checked that the upper voxel is not an infill and not a border voxel. If this was the case it means the point is part of the superior edge of an object and is moved up along the *Z*-Axis by the layer height. This new point cloud is called PC4. The Z-correction is displayed in Figure 4.

**Figure 5:**
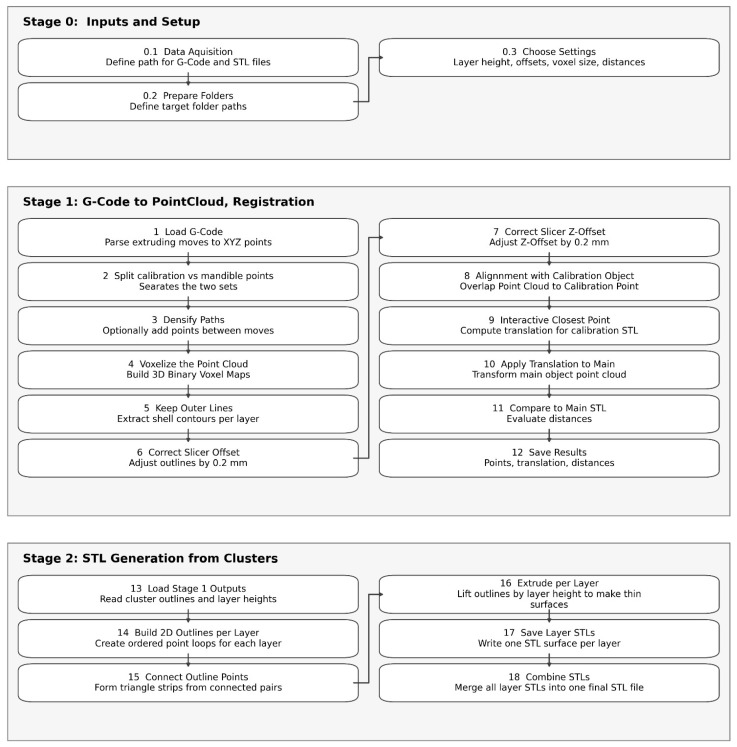
G-code analysis workflow. The process of analyzing G-code accuracy is segmented into three main phases: Stage 0 (Inputs and Setup) involves defining file paths and establishing general parameters; Stage 1 (G-code to Point-Cloud Registration) includes converting extrusion paths into XYZ coordinates, making adjustments in the XY and Z dimensions, and aligning with the calibration object; and Stage 2 (STL Generation from Clusters) is dedicated to reconstructing the 3D model by connecting layer contours and merging surfaces layer by layer. This workflow outlines the computational steps used to assess accuracy across all slicing software.

Figure 5 summarizes the computational workflow used to convert G-code into registered point clouds and STL models, including preprocessing, offset correction, and alignment steps.

### 2.6. G-Code to STL

**Figure 6:**
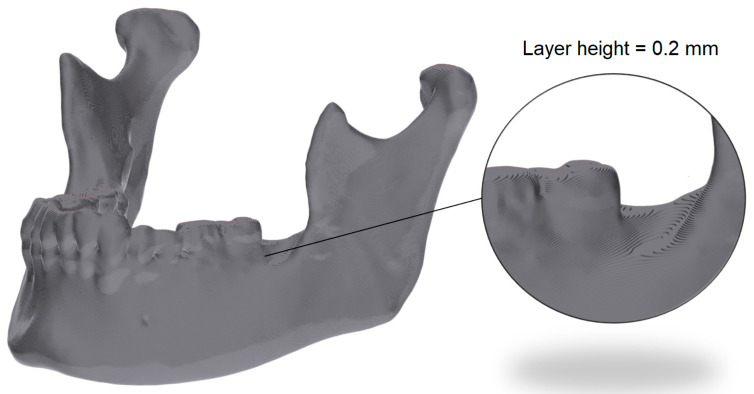
Volumetric reconstruction of a mandible from G-code data, generated by layer-wise extrusion of the toolpath-derived surface. The inset highlights the characteristic step-wise layering produced by the 3D printing process, accurately captured during STL conversion.

In order to perform a volumetric comparison, the previously calculated point cloud (PC4) was transformed into an STL object. Because all slicers were configured with a fixed layer height of 0.20 mm, the slicer-derived value extracted from each G-code file was identical. The volumetric reconstruction therefore used this same 0.20 mm height for all layers, ensuring consistency across slicers. The resulting volumes were merged sequentially to reproduce the layer-by-layer deposition process. Within each individual layer, all points were identified and a 2D surface was generated by connecting each point with its previous and following points defined in the G-code. This generated surface was then extruded by the slicer-defined layer height of 0.2 mm in +Z direction, simulating the nominal deposition step. This process was repeated for every layer and the resulting extruded volumes were combined into a single mesh. The volumetric reconstruction is displayed in Figure 6.

### 2.7. Calibration Object

Due to the fact that every slicer uses an own coordinate system, it was necessary to register the sliced objects to the original mesh. In order to avoid adding any inaccuracies, a calibration object (CO) was added to each mandible mesh before the slicing process. This newly generated mesh was sliced together with the mandible. Control tests confirmed that including the calibration object did not alter perimeter sequencing or infill generation of the mandibular model. Toolpaths generated with and without the calibration object were visually identical, and MSD differences were <0.005 mm.

After slicing, a KD-tree method was adopted, in order to subdivide the points of the point cloud into two different ones: PCM (point cloud mandible; contains the points of the G-code defining the mandible) and PCO (point cloud calibration object; contains the points of the G-code defining the calibration object). The PCO was corrected (XY-correction and Z-correction, as explained in Section 2.4 and Section 2.5). The center of mass of the corrected PCO was first translated onto the center of mass point of the calibration object. Subsequently, a rigid Iterative Closest Point (ICP) registration (translation + rotation, no scaling; 50 iterations; convergence < 0.02 mm) was applied to refine the alignment between the corrected PCO and the reference CO. The resulting transformation matrix was then applied to the PCM, yielding the translated point-cloud mandible (TPCM) aligned with the original STL reference.

### 2.8. Assessments

#### 2.8.1. Point Cloud Assessment

In order to assess the point clouds to each other, for each point of TPCM, the closest distance to the surface of the original mesh was determined. Subsequently, the mean surface distance (MSD), root mean square (RMS), minimum (Min), and maximum (Max) values of these distances were determined. MSD and RMS were chosen because the STL serves as the ground-truth reference, making unidirectional deviation the most interpretable measure. While symmetric metrics such as Hausdorff distance capture extreme outliers, they are less stable for voxel-derived surfaces and were therefore not used. The formulas for these metrics are displayed in Table 2.

Although voxelization uses a 0.10 mm grid, MSD is computed from the continuous corrected point cloud rather than from voxel centers. The voxel grid serves only to identify shell voxels, not to define comparison geometry. Therefore, deviations smaller than the voxel size can still be measured, and exploratory tests at 0.05 mm confirmed MSD stability (<0.005 mm change).

#### 2.8.2. Volumetric Assessment

In order to assess volumetric differences, the STL generated from the G-code was compared to the original mesh by comparing the volume of the meshes to one another and to determine the volume difference and the relative volume difference.

### 2.9. Accuracy Measurements

#### 2.9.1. Registration Accuracy Measurement

In order to assess the accuracy of the registration with the calibration object, the first mandible was sliced five times at the coordinates [X:0.0, Y:0.0, Z:0.0], [X:0.0, Y:100.0, Z:0.0], [X:100.0, Y:100.0, Z:0.0], [X:100.0, Y:100.0, Z:0.0], and [X:50.0, Y:50.0, Z:0.0] in Simplify3D, the only software where the coordinate system of the slicer corresponds to the coordinate system of the STL. For each G-code all points were corrected in XY- and Z-direction. Subsequently, the translation was found by registering the point cloud of the calibration object onto the STL, as specified in Section 2.7. In the second case the true translation was determined by extracting the coordinates at which the object was placed before slicing. Finally, the true and the calculated translation were applied on the mandible point cloud and the point cloud assessment was performed. The true and the calculated translation were compared to determine the inaccuracy of the registration. The mean distance between the mandibular point cloud and the original STL object was determined to assess the impact of the translation inaccuracy on the point cloud comparison.

#### 2.9.2. Slicer Repeatability Measurement

For each slicer the first mandible of the dataset was sliced 5 times at different positions in order to check the repeatability of the slicing process and assess its influence on the point cloud comparison. Each G-code was corrected in XY- and Z-direction, registered and the point cloud was compared to the original STL.

#### 2.9.3. Slicer Accuracy

In order to compare the accuracy of each slicer, all 20 mandibles of the dataset were sliced in each slicer and the G-code corrected in XY- and Z-direction, registered and the point cloud assessed to the original STL.

### 2.10. Statistical Analysis

To assess whether the results of the slicer evaluations follow a normal distribution, the Shapiro–Wilk test was applied to all numerical metrics. Because the data deviated from normality (*p* < 0.05), comparisons between slicers were performed with the non-parametric Kruskal–Wallis test. Post hoc pairwise comparisons were carried out using Dunn’s test with Bonferroni correction. Data are presented as mean ± standard deviation unless otherwise specified. Statistical analyses were performed in Python 3.8.10 using the scipy.stats package, with a significance level of α = 0.05.

## 3. Results

The statistical analysis included the slicers PrusaSlicer, Cura, Simplify3D, Slic3r, and Fusion, evaluating the metrics mean surface distance (MSD), root mean square error (RMS), minimum, and maximum deviation. Across all slicers, RMS values consistently deviated from normality, and minimum values were also frequently non-normally distributed. These findings indicate that the assumption of normality is not met for several key metrics. Therefore, non-parametric statistical methods were used to ensure methodological robustness in comparing slicer performance.

A Kruskal–Wallis test was applied to each metric and revealed statistically significant differences across the five slicers (*p* < 0.005). To determine which slicers differed, Dunn’s post hoc test with Bonferroni correction was performed. For the MSD and RMS metrics, both PrusaSlicer and Fusion differed significantly from all other slicers, while Cura, Simplify3D, and Slic3r showed no significant differences among each other. For the minimum metric, PrusaSlicer differed significantly from Cura and Slic3r, while Fusion differed significantly from Cura, Simplify3D, and Slic3r. No significant differences were found between Cura, Simplify3D, and Slic3r. For the maximum metric, Fusion differed significantly from all other slicers, whereas no significant differences were found among the remaining slicers.

### 3.1. Registration Accuracy Results

**Figure 7:**
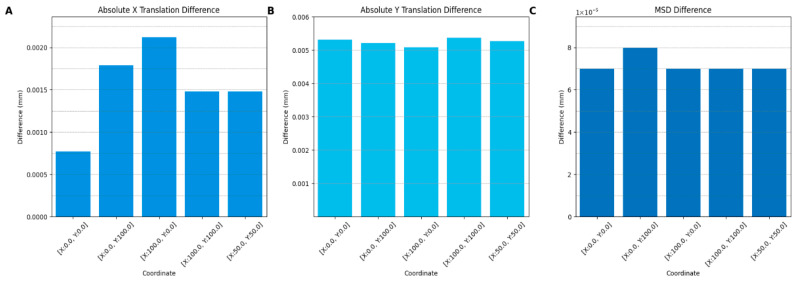
Evaluation of registration accuracy across multiple translations. (**A**) Absolute X translation difference between the real and the calculated translation. (**B**) Absolute Y translation difference between the real and the calculated translation. (**C**) Mean surface distance differences between the real and the calculated translation.

Based on the results of the registration accuracy measurement, it could be determined that the mean absolute translation in X and Y directions is 0.00153 mm and 0.00525 mm, respectively. These deviations in the X and Y direction resulted in an average difference of 0.000072 mm for the mean surface distance between the point cloud with the true translation applied in comparison to the actual translation. The results are displayed in Figure 7.

### 3.2. Slicer Repeatability Measurement Results

**Figure 8:**
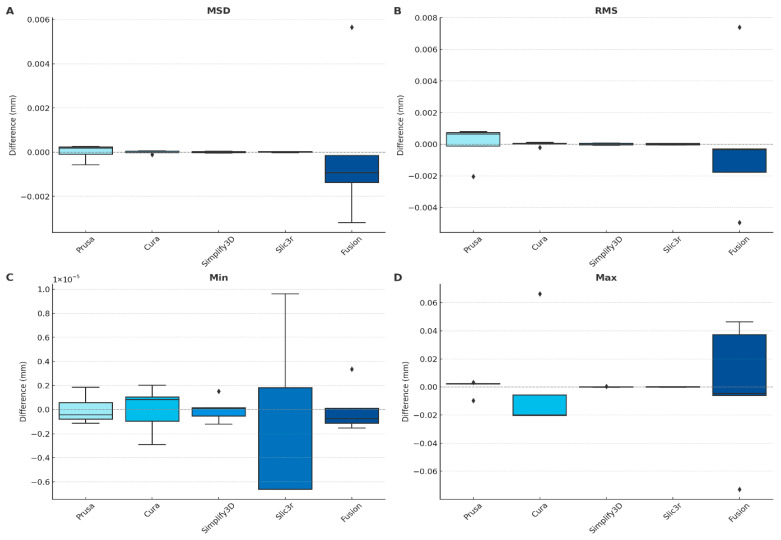
Assessment of slicer repeatability for different slicing engines (PrusaSlicer, Cura, Simplify3D, Slic3r, and Fusion) based on positional deviations relative to the original sliced mesh. (**A**) Mean surface distance. (**B**) Root mean square (RMS) deviation. (**C**) Minimum deviation. (**D**) Maximum deviation.

For each slicer the first mandible of the dataset was sliced 5 times at different positions in order to check the repeatability of the process. Each G-code was corrected in XY- and Z-direction, registered and the point cloud assessed to the original STL. The deviations to the average for the mean surface distance, the root mean square, the minimum and maximum distances were calculated. The results for each slicer are displayed in Figure 8.

### 3.3. Slicer Accuracy Results

**Figure 9:**
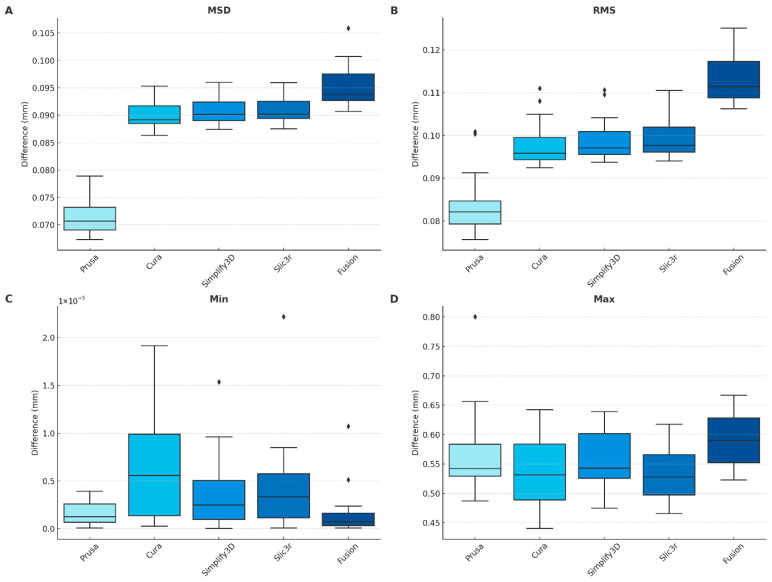
Assessment of slicer accuracy for different slicing engines (PrusaSlicer, Cura, Simplify3D, Slic3r, and Fusion) based on positional deviations relative to the original STL mesh across 20 distinct mandibular models. (**A**) Mean surface distance. (**B**) Root mean square (RMS) deviation. (**C**) Minimum deviation. (**D**) Maximum deviation.

To evaluate the accuracy of each slicer, all 20 mandibles in the dataset were sliced using PrusaSlicer, Cura, Simplify3D, Slic3r, and Fusion. For each slicer, the mean surface distance, root mean square (RMS) deviation, as well as the minimum and maximum distances were calculated to assess the positional accuracy. The summarized quantitative results are presented in Table 3, and the corresponding boxplots are shown in Figure 9. Among all slicers, PrusaSlicer showed the lowest mean surface distance (0.071 mm) and RMS deviation (0.084 mm). In contrast, Fusion exhibited the highest deviations, with a mean of 0.095 mm and RMS of 0.113 mm. The maximum surface deviations ranged from 0.532 mm (Slic3r) to 0.591 mm (Fusion). Across slicers, the largest deviations consistently occurred in regions of high curvature such as the coronoid process, mandibular notch, and lingual surface. This recurrence indicates algorithm-driven behavior rather than random toolpath noise.

The Kruskal–Wallis test revealed significant differences among slicers (MSD: H = 18.4, df = 4, *p* < 0.001, ε^2^ = 0.67; RMS: H = 16.9, df = 4, *p* < 0.001, ε^2^ = 0.62). Dunn–Bonferroni post hoc tests showed that Fusion differed significantly from all other slicers (*p* < 0.01). Effect sizes (Cliff’s δ) were computed for all pairwise comparisons and confirmed these findings, with Fusion exhibiting moderate-to-large effects (δ = 0.64–0.79) relative to the other engines. In contrast, Cura, Simplify3D, and Slic3r showed small or negligible effect sizes and no significant differences among themselves (*p* > 0.05). PrusaSlicer achieved the lowest median MSD (0.071 mm [0.012]) and RMS (0.084 mm [0.015]), followed by Cura (0.090/0.097 mm), Simplify3D (0.091/0.100 mm), Slic3r (0.091/0.098 mm), and Fusion (0.095/0.113 mm). Maximum surface deviations ranged from 0.532 mm (Slic3r) to 0.591 mm (Fusion), while minimum distances were near zero across slicers.

### 3.4. Volumetric Comparison

**Figure 10:**
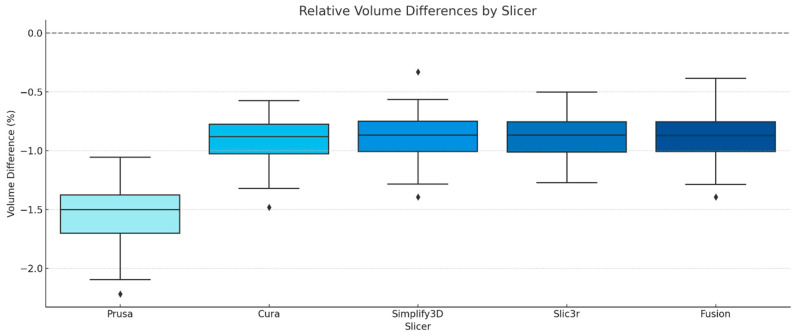
Relative volume differences across slicing engines (PrusaSlicer, Cura, Simplify3D, Slic3r, and Fusion) based on comparison with the original STL mesh across 20 distinct mandibular models.

To assess volumetric differences introduced by the slicing process, the STL files generated from the G-code were compared to the original reference mesh. The volumetric comparison was based on calculating the relative volume differences between the reconstructed meshes and the original STL. The results are summarized in Figure 10. On average, PrusaSlicer showed the largest relative volume deviation at −1.55% ± 0.32%, while Cura, Simplify3D, Slic3r, and Fusion exhibited smaller and nearly identical deviations around −0.89% to −0.93%, with standard deviations ranging from 0.22% to 0.26%.

## 4. Discussion

This study investigated the geometric and volumetric accuracy of G-code generated by five commonly used slicing software packages: PrusaSlicer, Cura, Simplify3D, Slic3r, and Fusion. The comparison was made between reconstructed models derived from the G-code and the original STL files. A custom Python-based pipeline was developed to extract point clouds from the G-code, apply geometric corrections, and compare them with the reference meshes. This allowed for precise assessment of slicing-induced deviations independently of printer hardware. The discussion below addresses the validity of the registration method, the repeatability of slicing outputs, the accuracy of the slicers across multiple models, and the implications for clinical use and process validation.

### 4.1. Registration Accuracy and Methodological Reliability

Before evaluating the slicing accuracy, the reliability of the registration process needed to be established. The results of the registration accuracy measurement showed that the error caused by the registration process is negligible. The measured mean absolute translation error was 0.00153 mm in the X direction and 0.00525 mm in the Y direction. These discrepancies resulted in a negligible mean surface distance (<0.001 mm), indicating that registration error was insignificant. This demonstrates that the calibration object-based registration technique used in this study is precise and introduces no relevant bias in the alignment of reconstructed G-code data with the original STL mesh. As a result, the deviations described in the following sections can confidently be attributed to the slicing process itself.

### 4.2. Repeatability of the Slicing Software

The consistency of each slicing software was assessed by generating five G-codes for the same mandible model, each with a distinct position on the virtual build platform. To quantify repeatability, deviations from the slicer’s own average values were calculated for each slicing run. The evaluation considered key geometric metrics, including mean surface distance, root mean square (RMS) deviation, minimum deviation, and maximum deviation.

Among the evaluated slicers, Simplify3D demonstrated the highest repeatability, with mean surface distance consistently remaining within ±0.00004 mm across all positional variations. Cura and Slic3r also exhibited stable performance, with low intra-slicer variability. PrusaSlicer showed greater internal fluctuation between slicing positions. Fusion displayed the lowest repeatability, with RMS deviations varying by up to 0.002 mm depending on the model’s placement within the slicing environment.

Even with all randomization features disabled, minor deviations were consistently detected between repeated slicing runs, confirming that a degree of non-deterministic behavior is intrinsic to the internal toolpath computation of most slicing engines. This phenomenon likely results from floating-point rounding and parallelized path-planning routines rather than user-controlled randomness. Consequently, positional sensitivity and small geometric fluctuations should be considered when defining quality-assurance thresholds for medical additive manufacturing.

### 4.3. Accuracy Across Multiple Anatomical Models

To evaluate the accuracy of each slicer, all twenty mandibles in the dataset were processed using PrusaSlicer, Cura, Simplify3D, Slic3r, and Fusion. The mean surface distance, root mean square (RMS) deviation, minimum, and maximum distances were calculated to assess the geometric accuracy. The lowest mean surface distance was obtained with PrusaSlicer (0.071 mm), followed by Cura (0.090 mm), Simplify3D (0.091 mm), Slic3r (0.091 mm), and Fusion (0.095 mm). RMS values ranged from 0.084 mm for PrusaSlicer to 0.113 mm for Fusion. Average maximum deviations varied between 0.532 mm for Slic3r and 0.591 mm for Fusion, with PrusaSlicer showing 0.565 mm despite its low average error. The minimum deviations for all slicers were close to zero.

Although PrusaSlicer achieved the lowest mean surface distance, its maximum deviations were not proportionally reduced. For example, PrusaSlicer had the best average accuracy but one of the highest maximum deviations. This indicates that local outliers can occur even in generally accurate toolpaths and underlines the importance of considering both global and peak errors when evaluating slicing accuracy. Although mean deviations were similar across several slicers, maximum deviations diverged substantially, reflecting localized geometric artifacts such as corner rounding and perimeter merging strategies that disproportionately affect peak error metrics. Regions with high curvature or thin anatomical ridges exhibited greater slicer-induced deviation, consistent with known differences in how slicing engines approximate curved perimeters and apply offsetting strategies in concave areas.

### 4.4. Volumetric Accuracy and Relevance for Process Validation

In addition to surface-based comparisons, this study also evaluated volumetric accuracy by reconstructing STL meshes from the corrected G-code and comparing the resulting volumes to the original reference models. The relative volume difference was calculated for each slicing engine.

All slicers produced a consistent negative volume deviation relative to the reference STL models. In every case, the calculated volumes were smaller than the original STL volumes, indicating negative deviations introduced during the slicing process (Table 3). A significant divergence was observed when comparing results to the surface accuracy data. PrusaSlicer, which yielded the best surface accuracy, paradoxically exhibited the largest average negative volume deviation, with a mean volume difference of −1.55% ±0.32% and a range from −2.22 to −1.06%. This pattern indicates that PrusaSlicer’s tool-path strategy may favor close surface conformity at the expense of overall volume. In contrast, Simplify3D, Slic3r, Fusion, and Cura showed smaller but still relevant deviations. PrusaSlicer’s volumetric underestimation likely results from aggressive perimeter overlap and internal trimming strategies that maintain excellent surface conformity but slightly reduce enclosed volume, explaining why surface accuracy was highest while volumetric accuracy was lowest. Simplify3D demonstrated the smallest average volumetric error, with a mean volume difference of −0.89 ±0.35% and a minimum deviation of −0.33%. Slic3r and Fusion showed similar volumetric values, with mean deviations of −0.89% and −0.90%, respectively. Cura yielded a mean deviation of −0.93%, slightly higher than that of Simplify3D.

These findings indicate that the slicing process contributes significantly to the volumetric deviation observed in printed models. If such deviations are already present in the G-code, they will be replicated by the printer regardless of its mechanical precision. These findings highlight the value of assessing G-code output when evaluating dimensional accuracy. In cases where general-purpose slicers are used and slicing parameters are not tightly controlled, the volumetric accuracy of the final print may be compromised. For clinical or regulatory applications that require high dimensional fidelity, especially where volume preservation is relevant, verification of the slicing output is advised for a reliable process validation.

### 4.5. Importance for Clinical Use and Regulatory Approval

This study quantifies slicer-induced geometric deviations by comparing G-code derived geometries with their reference STL meshes. The developed method enables conversion of G-code into a point cloud and an STL model for accurate comparison. It was found that mean surface distances between the original mesh and the reconstructed G-code geometry lie between 0.077 and 0.103 mm, depending on the slicer. These values are clinically relevant, particularly when compared to the total printing errors typically reported for fused deposition modeling. The literature values for root mean square errors in FDM printing range from 0.15 to 0.16 mm [7]. By comparing these findings with the RMS found for the slicer, ranging from 0.09–0.13 mm, depending on the utilized slicer, it is observable that the slicer`s error determined in this study is responsible for more than 50% of the full process error. Further studies should investigate how improved G-code precision translates into physical printing accuracy. Although this study does not evaluate clinical devices, the slicer-induced deviations observed here (0.07–0.10 mm) represent a non-negligible dimensional error relative to the 0.1–0.5 mm tolerances commonly reported for anatomical models. Thermal distortion in FDM printing typically introduces RMS errors of 0.10–0.25 mm, placing slicer-related deviations in a comparable range and identifying them as an inherent upstream error source that cannot be mitigated through hardware calibration.

In clinical and regulatory settings, where process validation and dimensional traceability are essential, the accuracy of the G-code must be verified. Medical devices such as surgical models, templates, and implants require not only accurate printing but also a validated digital workflow. Demonstrating that the G-code is a faithful and accurate transformation of the STL model is a necessary step in this chain. The method presented in this study can be directly used for this purpose and thus has significant potential to improve quality control protocols in hospital-based additive manufacturing.

Furthermore, the pipeline developed here does not only serve verification purposes. Having the ability to extract, analyze, and modify G-code opens new opportunities in toolpath correction, extrusion compensation, or intelligent path planning. This capability may support applications such as toolpath correction, extrusion compensation, and customized motion planning.

### 4.6. Limitations and Outlook

While the method developed and validated in this study is robust, several limitations must be acknowledged. The calibration object must remain geometrically intact in the G-code. Although registration is robust to minor segmentation of its perimeters, substantial occlusion or fragmentation could degrade alignment accuracy.

The analysis was restricted to mandibular models and may not be directly applicable to more complex or hollow structures. Additionally, the current version of the Python tool can only process solid, watertight objects and does not yet support models with internal voids.

The reconstruction assumes a uniform line width as defined in the slicer configuration. Although actual printed line widths may vary with flow rate and speed, this study isolates slicer-induced geometric deviations at the G-code level, where the nominal line width is explicitly defined. Consequently, the analysis evaluates slicer geometry rather than physical extrusion behavior.

This study focuses exclusively on slicer-induced geometric deviations and therefore does not include a factorial analysis of design or process parameters. Such parameters strongly affect the accuracy of physical prints, but incorporating them would obscure the slicer-specific effects that this study aims to isolate.

The XY-offset model assumes circular filament expansion based on the user-defined line width. While real FDM filaments are often elliptical, this study evaluates slicer-intended geometry rather than physical deposition, and all slicers internally use the nominal line width for perimeter generation.

Adaptive layer heights were disabled because they introduce geometry-dependent Z-variation that is not consistent across slicers and would confound cross-slicer comparisons. Future studies may evaluate slicer-induced deviation under adaptive-height conditions.

Future development should include support for hollow or internally complex geometries, as the current findings may not generalize to thin-walled anatomical structures (e.g., sinus or orbital floors), where perimeter offsetting and minimum-wall constraints are likely to induce greater slicer-induced deviations than those observed in the analyzed mandibular models.

Finally, this study did not include the physical printing step. While the focus here was to isolate slicer-induced errors, validation against physical prints was not performed and should be included in future studies.

## 5. Conclusions

This study presents a detailed evaluation of slicing software accuracy by comparing G-code-based reconstructions with original STL models. The results show that slicers introduce measurable deviations in surface geometry and volume, even under standardized conditions. Among the five tested, PrusaSlicer achieved the best surface accuracy (lowest MSD/RMS), while Simplify3D and Slic3r demonstrated superior volumetric fidelity. Simplify3D was also found to have the highest repeatability, and Fusion consistently exhibited the largest overall deviations. A key finding is that slicer-induced deviations contribute to the overall error and should be considered when assessing printing accuracy. If the G-code contains errors, the printer will reproduce them precisely. This is especially relevant for printers that rely on external slicers, where user settings can affect print quality. The proposed method may be relevant for clinical and regulatory validation, where demonstrating G-code precision is crucial.

In conclusion, this work identifies an often-overlooked source of error in 3D printing and offers a practical tool to improve accuracy in medical additive manufacturing.

## Figures and Tables

**Table 1:** Decision logic for point correction in the XY-plane based on infill detection.

P′	P″	Points Added	Meaning
P′ in infill	P″ in infill	None	Error
P′ in infill	P″ not in infill	P″ considered	Correct point found
P′ not in infill	P″ in infill	P′ considered	Correct point found
P′ not in infill	P″ not in infill	P′ and P″ considered	Single line

**Table 2:** List of the metrics used in this study with their corresponding formulas and descriptions.

Metric	Formula	Legend
Mean Surface Distance (MSD)	$\mathrm{Signed}\;\mathrm{MSD}=\frac{1}{n_{A}}\left(\sum_{i=1}^{n_{A}}\;\;\underset{p\in P}{min}\;\left\|a_{i}-p\right\|\cdot sign\left(\left(a_{i}-p\right)\cdot n_{p}\right)\right)$	The average distance from all points of point cloud A to their closest points on point cloud P.
Root Mean Square (RMS)	$RMS=\sqrt{\frac{1}{n_{A}}\sum_{i=1}^{n_{A}}\;\;{\left(\underset{p\in P}{min}\;\left\|a_{i}-p\right\|\right)}^{2}}$	The square root of the average of the squared signed distances from all points of point cloud A to their closest points on point cloud P.
Minimal (Min)	$d_{\min,\;\mathrm{signed}\;}=\underset{i=1}{\overset{n_{A}}{min}}\;\left(\underset{p\in P}{min}\;\left(\left\|a_{i}-p\right\|\cdot sign\left(\left(a_{i}-p\right)\cdot n_{p}\right)\right)\right)$	The smallest distance from any point of point cloud A to their closest points on point cloud P.
Maximal (Max)	$d_{\max,\;\mathrm{signed}\;}=\underset{i=1}{\overset{n_{A}}{max}}\;\left(\underset{p\in P}{min}\;\left(\left\|a_{i}-p\right\|\cdot sign\left(\left(a_{i}-p\right)\cdot n_{p}\right)\right)\right)$	The largest distance from any point of point cloud A to their closest points on point cloud P.

**Table 3:** Comparative slicer accuracy across 20 mandibles (median [IQR]). Δ Volume is the relative volumetric difference between the reconstructed mesh and the reference STL.

Slicer	MSD [mm] Median [IQR]	RMS [mm] Median [IQR]	Min [mm]	Max [mm]	Δ Volume [%] Median [IQR]
PrusaSlicer	0.071 [0.012]	0.084 [0.015]	0.028	0.131	−1.55 [0.32]
Cura	0.090 [0.014]	0.097 [0.016]	0.033	0.144	−0.93 [0.26]
Simplify3D	0.091 [0.015]	0.100 [0.017]	0.037	0.150	−0.89 [0.22]
Slic3r	0.091 [0.013]	0.098 [0.016]	0.035	0.152	−0.89 [0.25]
Fusion 360	0.095 [0.016]	0.113 [0.018]	0.041	0.164	−0.90 [0.23]

## Data Availability

The original contributions presented in this study are included in the article. Further inquiries can be directed to the corresponding author.

## Abbreviations

The following abbreviations are used in this manuscript:

3D	Three-Dimensional
CT	Computed Tomography
DICOM	Digital Imaging and Communications in Medicine
FDM	Fused Deposition Modeling
MSD	Mean Surface Distance
RMS	Root Mean Square
STL	Standard Tessellation Language
